# Quantification of cortisol and its metabolites in human urine by LC-MS^n^: applications in clinical diagnosis and anti-doping control

**DOI:** 10.1007/s00216-022-04249-3

**Published:** 2022-08-02

**Authors:** Francesco Arioli, Maria Cristina Gamberini, Radmila Pavlovic, Federica Di Cesare, Susanna Draghi, Giulia Bussei, Francesca Mungiguerra, Alessio Casati, Marco Fidani

**Affiliations:** 1 Department of Veterinary Medicine and Animal Science, University of Milan, Via dell’Università 6, 26900 Lodi, LO Italy; 2 Department of Life Science, University of Modena and Reggio Emilia, Via Campi 103, 41125 Modena, Italy; 3 UNIRELAB Srl, Via Gramsci 70, 20019 Settimo Milanese, MI Italy

**Keywords:** Linear ion trap mass spectrometry, Cortisol metabolites, Human urine, Addison syndrome, Cushing syndrome, Doping control

## Abstract

The objective of the current research was to develop a liquid chromatography-MS^n^ (LC-MS^n^) methodology for the determination of free cortisol and its 15 endogenous metabolites (6β-hydroxycortisol, 20α-dihydrocortisol, 20α-dihydrocortisone, 20-β-dihydrocortisol, 20β-dihydrocortisone, prednisolone, cortisone, α-cortolone, β-cortolone, allotetrahydrocortisol, 5α-dihydrocortisol, tetrahydrocortisol, allotetrahydrocortisone, 5β-dihydrocortisol, tetrahydrocortisone) in human urine. Due to its optimal performance, a linear ion trap operating in ESI negative ion mode was chosen for the spectrometric analysis, performing MS^3^ and MS^4^ experiments. The method was validated for limit of detection (LOD) and limit of quantification (LOQ) (0.01 ng mL^−1^ and 0.05 ng mL^−1^, for all compounds, respectively), intra- and inter-day precision (CV = 1.4–9.2% and CV = 3.6–10.4%, respectively), intra- and inter-day accuracy (95–110%), extraction recovery (65–95%), linearity (R2 > 0.995), and matrix effect that was absent for all molecules. Additionally, for each compound, the percentage of glucuronated conjugates was estimated. The method was successfully applied to the urine (2 mL) of 50 healthy subjects (25 males, 25 females). It was also successfully employed on urine samples of two patients with Cushing syndrome and one with Addison’s disease. This analytical approach could be more appropriate than commonly used determination of urinary free cortisol collected in 24-h urine. The possibility of considering the differences and relationship between cortisol and its metabolites allows analytical problems related to quantitative analysis of cortisol alone to be overcome. Furthermore, the developed method has been demonstrated as efficient for antidoping control regarding the potential abuse of corticosteroids, which could interfere with the cortisol metabolism, due to negative feedback on the hypothalamus-hypophysis-adrenal axis. Lastly, this method was found to be suitable for the follow-up of prednisolone that was particularly important considering its pseudo-endogenous origin and correlation with cortisol metabolism.

**Graphical abstract:**

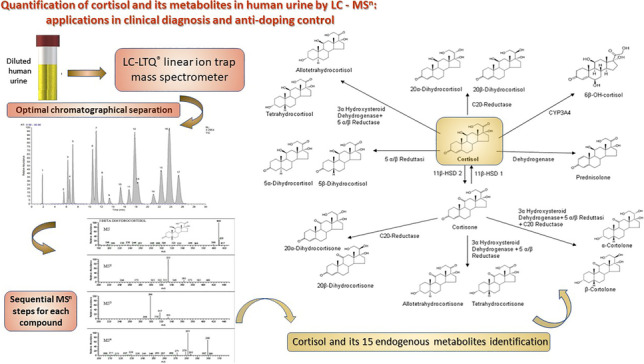

## Introduction

Cortisol is a steroid hormone synthesized in the adrenal cortex, involved in glucidic metabolism, lipidic metabolism, and anti-inflammatory and immunosuppressive activity. Its secretion is controlled, through the hypothalamus-hypophysis-adrenal axis, by the corticotropin-releasing hormone (CRH) and adrenocorticotropic hormone (ACTH), and by cortisol itself that acts with a negative feedback mechanism on its secretion [1].

**Fig. 1:**
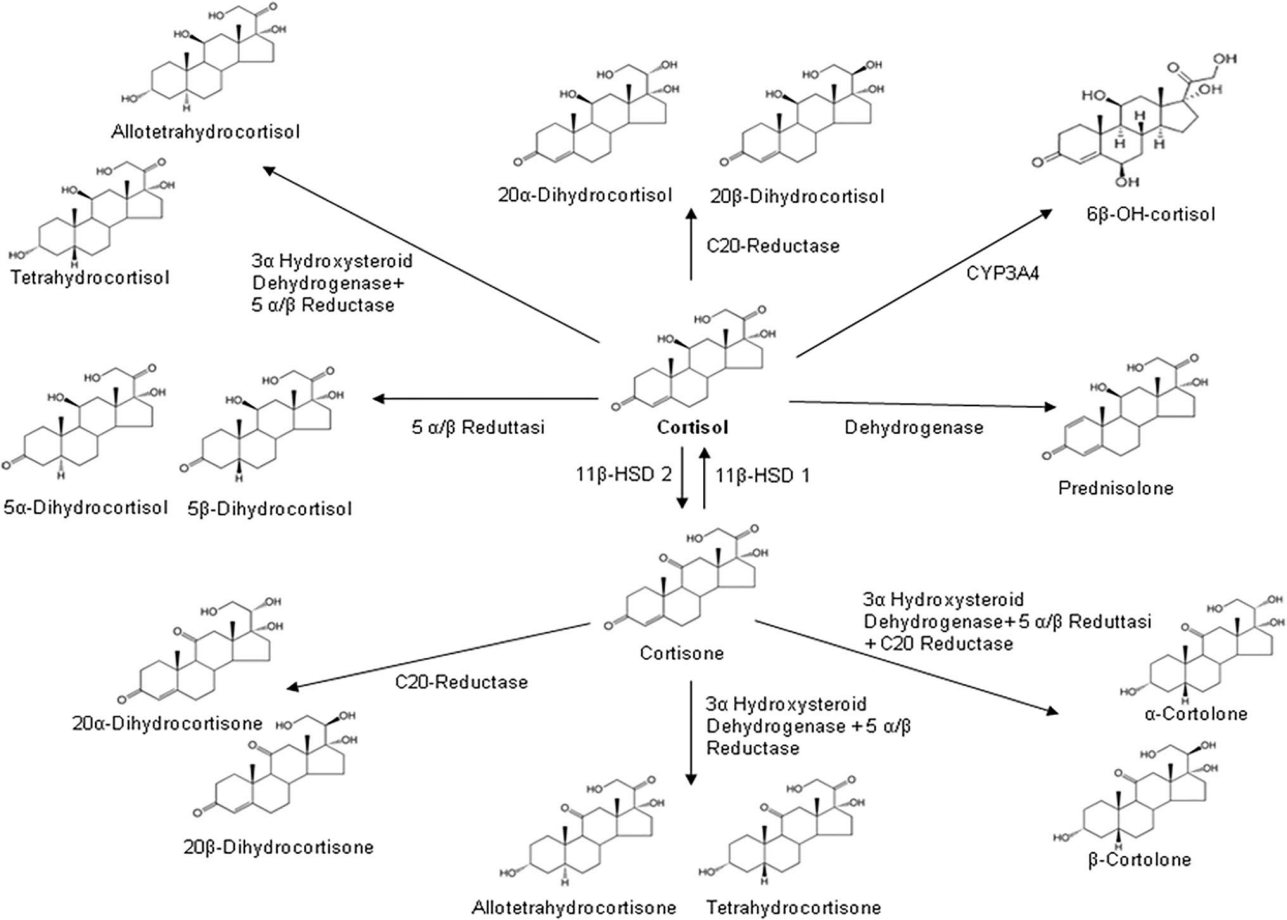
Chemical structures of cortisol and metabolites investigated

Cortisol is metabolized by the liver and kidneys. The main enzymes involved in phase 1 metabolic reactions are 11β-hydroxysteroid dehydrogenase isoform 1 (11β-HSD 1) that converts cortisone in cortisol and 11β-hydroxysteroid dehydrogenase isoform 2 (11β-HSD 2) that oxides cortisol in its inactive metabolite cortisone. Other enzymes with reductase and dehydrogenase activity allow the formation of other urinary metabolites. The most important are 5 α/β reductase, 3 α/β hydroxysteroid dehydrogenase, C20 reductase, and CYP3A4 [2, 3]. Interconversion of cortisol/cortisone by 11β-HSDs and formation of their A ring reduced metabolites (tetrahydrocortisol, allo-tetrahydrocortisol, tetrahydrocortisone, and allo-tetrahydrocortisone) as well as corresponding 20β-, 5β- 6β hydroxy metabolites are presented in Fig. 1. Furthermore, the correlation between cortisol/prednisolone must be taken into consideration as one of the important metabolic modifications of cortisol [4]. The metabolism of glucocorticoids includes also phase II conjugation reactions principally with glucuronic acid through UDP-glucuronyltransferase (UDP-GT) enzyme.

For their importance in the metabolic processes, many analytical methods have been developed for the detection and measurement of endogenous corticosteroids in human samples [5, 6]. In clinical analysis, cortisol measurement is used in the diagnosis of different endocrine disorders like Cushing’s syndrome, Addison disease, adrenal mineralocorticoid excess syndrome, congenital adrenal hyperplasia, adrenocortical cancer, and adrenal cortical adenoma [6, 7].

In these diagnoses, the three major tests performed are the measurement of urinary free cortisol in 24 h, plasma cortisol, and plasma ACTH [8]. Routine determination of cortisol is commonly performed by immunoassay tests, easy to perform, but lacking specificity due to the cross-reactivity problem of the antibodies used [9, 10]. Indeed, in human matrices, the presence of compounds structurally similar to cortisol is not negligible. Also, the presence of exogenous glucocorticoids, used for therapy, can give interference with the quantification of cortisol itself.

The extreme sensitivity and specificity of mass spectrometry can solve these problems [11]. In particular, for corticosteroid analysis in clinical diagnosis, different gas chromatography-mass spectrometry (GC–MS) methods were developed [12, 13], but, because of low corticosteroid volatilities, a derivatization step was always needed, so the throughput of a routine process can be difficult.

These problems were solved with the introduction of liquid chromatography-mass spectrometry (LC–MS) analysis that provides high analytical sensitivity and specificity, and rapid sample preparation [9, 14–26]. Thanks to these advantages, different LC–MS methods were proposed in the diagnosis of pathologies in which endogenous corticosteroids can be involved [27–31]. It has largely been demonstrated that LC–MS is the analytical method of choice for endogenous and exogenous corticosteroid measurements in human samples [32].

For LC–MS analysis of corticosteroids, triple quadrupole mass spectrometry operating in multiple reaction monitoring (MRM) in positive ion mode with electrospray source (ESI) is the most frequently used method to analyze these molecules [27–29]. On the contrary, there is just a few publications that deal with ion trap mass detector application in cortisol determination [33, 34]. Furthermore, information that regards the usage of LC-MS^n^ methodology in cortisol metabolic profiling is lacking in the available literature. Therefore, this study was designed to develop and validate a LC-MS^n^ method performed in electrospray (ESI) negative ion mode, for the qualitative and quantitative analyses of cortisol and 15 metabolites in human urine. Subsequently, the practical utility of this analytical platform for diagnostic and antidoping purposes will be preliminarily investigated.

## Materials and methods

### Reagents and chemicals

Cortisol [4-pregnen-11β,17α,21-triol-3,20-dione], prednisolone [1,4-pregnadiene-11β,17α,21-triol-3,20-dione], 6β-hydroxycortisol [4-pregnen-6β,11β,17,21-tetrol-3,20-dione], cortisone [17α,21-dihydroxy-4-pregnene-3,11,20-trione], allotetrahydrocortisol [5α-pregnan-3α,11β,17α,21-tetrol-20-one], tetrahydrocortisol [5β-pregnan-3α,11β,17α,21-tetrol-20-one], allotethrahydrocortisone [5α-pregnan-3α,17α,21-triol-11,20-dione], tetrahydrocortisone [5β-pregnan-3α,17α,21-triol-11,20-dione], 20α-dihydrocortisol [4-pregnen-11β,17α,20α,21-tetrol-3-one], 20-β dihydrocortisol [4-pregnen-11β,17α,20β,21-tetrol-3-one], 5α-dihydrocortisol [5α pregnan-11β,17α,21-triol-3,20-dione], 5β-dihydrocortisol [5β-pregnan-11β,17α,21-triol-3,20-dione], α-cortolone [5β-pregnan-3α,17α,20α,21-tetrol-11-one], β-cortolone [5β-pregnan-3α,17α,20β,21-tetrol-11-one], 20α-dihydrocortisone [4-pregnen-17α, 20α,21-triol-3,11-dione], 20β-dihydrocortisone [4-pregnen-17α,20β,21-triol-3,11-dione], and 6α-methylprednisolone [11β,17α,21-trihydroxy-6α-methyl-1,4-pregnadiene-3,20-dione] as internal standard (IS) were from Steraloids (Newport, RI, USA). β-Glucuronidase (*E. coli* K12 243 U mg^−1^) was from Roche (Mannheim, Germany).

All solvents were of HPLC grade. *Tert*-butyl methyl ether, acetonitrile, formic acid, and ammonium acetate, were from Sigma-Aldrich (St. Louis, MO, USA). Water was freshly prepared with a Milli-Q Advantage A10 Ultrapure Water Purification System (Merck-Millipore, Darmstadt, Germany).

Standard stock solutions (1 mg mL^−1^) were prepared by dissolving the dry powder of each analyte in methanol; solutions were stored at − 20 °C. Working solutions were prepared daily by diluting the stock solutions with methanol.

### Sample collection

The concentrations of free cortisol and 15 free metabolites were calculated in the urine of 50 healthy volunteers (25 males and 25 females, 20 to 70 years old) divided into 5 groups of 10 people each (5 men and 5 women). Each group was subjected to a single urine collection at a given time of the day, i.e., 7 a.m.–11 a.m., 11 a.m.–15 p.m., 15 p.m.–19 p.m., 19 p.m.–23 p.m., and 23 p.m.–7 a.m. The 24-h urine was also collected from two volunteers (one male and one female).

### Sample preparation

Two milliliters of human urine was spiked with 50 μL of the internal standard methylprednisolone to a final concentration of 2.5 ng mL^−1^. Tert-butyl methyl ether (2 mL) was added. After shaking in a vertical rotary shaker for 20 min, the sample was centrifuged at 2000 g for 20 min.

The upper layer was collected with a Pasteur pipette, transferred into a 10-mL glass tube, and dried under nitrogen stream at room temperature. The residue was dissolved in 100 µL of the mobile phase (23% acetonitrile, 77% aqueous solution 0.1% formic acid) and injected into the LC–MS system. The injection volume was 5 µL.

For all the compounds, to have a percentage value of the glucuronic acid derivatives of each compound, a hydrolysis step with β-glucuronidase was performed on 20 urine samples (10 males, 10 females) in which the free-form concentrations of every compound had been previously detected without hydrolysis.

For the hydrolysis procedure, 500 µL of ammonium acetate buffer 1 M (pH = 6.1) and 40 μL of β-glucuronidase were added. Samples were incubated for 60 min at 55 °C ± 5 °C. The extraction steps were the same as described for samples not undergoing deconjugation.

### LC–MS parameters

The chromatographic separation was performed at room temperature, in isocratic condition, on a reversed-phase C18 Sunfire® column (150 × 2.1 mm i.d., 3.5 µm particle size; Waters, Milford, MA, USA) equipped with a Sunfire C18 Guard Column® (2.1 × 10 mm i.d., 3.5 µm particle size; Waters, Milford, MA, USA). The mobile phase consisted of a mixture of aqueous solution 0.1% formic acid and acetonitrile (77:23) at a flow rate of 0.3 mL min^−1^.

An LTQ® linear ion trap mass spectrometer equipped with an Electrospray Source (Thermo Fisher, San José, CA, USA), connected to a Surveyor Autosampler and a Surveyor MS Pump (Thermo Fisher, San José, CA, USA), was used.

The linear ion trap was operated in negative electrospray ionization mode [ESI ( −)] under the following conditions: sheath and auxiliary gas (nitrogen) flow rates of 40 and 20 arbitrary units, respectively; sweep gas was off; spray voltage of 4 kV; ion transfer capillary temperature 275 °C; capillary voltage − 5 V; and tube lens − 30 V. Helium was used as collision gas. Collision energy ranged between 18 and 25%.

Due to the fact that all compounds considered in the study can form a very abundant and stable adduct with formic acid in ESI ( −) mode ([M + HCOO]^−^), the MS parameters were optimized using the cortisol adduct with formic acid (MW = 407 Da) as reference compound, by direct injection of a standard solution of cortisol (1 µg mL^−1^) at a flow rate of 20 µL min^−1^. Data acquisition and analysis were accomplished using Xcalibur® software version 2.1 (Thermo Fisher, San José, CA, USA).

The compounds, their retention times, their molecular weights, the molecular weights of their adducts with formic acid (used as precursor ions), and the product ions with the related molecular weight are shown in Table 1, together with the collision energies (CE%). The product ions obtained after the MS^2^ experiment were used as precursor ions for the subsequent MS^3^ and eventual MS^4^ steps of analysis.

**Table 1:** Corticosteroids with their molecular weights (MW), retention times (Rt), m/z values of their adducts with formic acid (used as precursor ions for MS^2^ fragmentation), collision energy (CE%) for MS^2^ fragmentation, product ion in the MS^2^ spectrum used like precursor ion for MS^3^ fragmentation, collision energy (CE%) for MS^3^ fragmentation, product ions in the MS^3^ spectrum with underlined quantitative ion used like precursor ion for MS^4^ fragmentation, collision energy (CE%) for MS^4^ fragmentation, and product ions in the MS^4^ spectrum

Corticosteroid	MW	Rt	Precursor ion [M − HCOO]^−^	CE%	Product ion MS^2^	CE%	Product ions MS^3^	CE%	Product ion MS^4^
6β-Hydroxicortisol	378	1.8	423	20	347	20	205, 313, 331	–	–
20α-Dihydrocortisol	364	5.5	409	20	363	20	333	20	273, 315
20α-Dihydrocortisone	362	6.2	407	20	361	20	331	25	245, 271, 315
20β-Dihydrocortisol	364	6.5	409	20	363	20	333	20	273, 297, 315
20β-Dihydrocortisone	362	7.1	407	20	361	20	331	25	245, 271, 315
Prednisolone	360	10.5	405	20	329	20	280, 295, 313	–	–
Cortisol	362	11.1	407	20	331	20	189, 297, 315	–	–
Cortisone	360	12.1	405	20	329	20	301, 311	–	–
α-Cortolone	366	13.4	411	20	365	20	275, 335	23	247, 275, 299
β-Cortolone	366	15.3	411	20	365	20	335	23	247, 275, 299
Allo-tetrahydrocortisol	366	16.8	411	20	335	20	301, 319	–	–
5α-Dihydrocortisol	364	17.8	409	20	333	18	299, 317	25	279, 281
Tetrahydrocortisol	366	18.3	411	20	335	20	301, 319	–	–
Methylprednisolone (IS)	374	21	419	20	343	20	294, 309, 327	–	–
Allo-tetrahydrocortisone	364	22.4	409	20	333	18	305	25	209, 261, 287
5β-Dihydrocortisol	364	23.8	409	20	333	18	299, 317	25	279, 281
Tetrahydrocortisone	364	25.4	409	20	333	18	305	25	209, 261, 287

### Method validation

Due to the endogenous nature of the corticosteroids involved in the study, an analyte-free matrix (blank) was obtained from a volunteer treated with a synthetic glucorticosteroid by oral administration (betamethasone, 1 mg day^−1^ for 7 days). On the fourth day after first administration, cortisol and consequently all its metabolites were lower than the LOD, for the effect of the negative feedback of the synthetic glucorticosteroid on the hypothalamus-hypophysis-adrenal axis. Urine collected after this time was used as blank urine to calculate all the validation parameters of the method. Validation was performed following the WADA guideline with more details provided in Eurachem guide [35–37]. For each analyte, the method performance was assessed through (1) qualitative parameters achieved by specificity and by the identification of compounds in urine with respect to the retention time and fragmentation pattern of their analytical standards; (2) quantitative parameters, such as the linearity, accuracy in terms of trueness (valued as bias), and precision expressed as the intra- and inter-day repeatability; (3) analytical sensitivity estimated as limit of detection (LOD) and limit of quantification (LOQ). Additionally, the robustness of the method as well as matrix effect was assessed, too.

For the linearity, two calibration curves were prepared on blank urine depending on the concentrations expected for each compound in healthy human urine [17], preliminarily calculated with a semiquantitative approach. The first calibration curve was prepared with six “low” concentrations of the free forms (0.05, 0.10, 0.25, 0.5, 1, 5 ng mL^−1^) while six “high” concentrations were used for the second calibration curve (5, 10, 50, 100, 250, 500 ng mL^−1^). Two curves were prepared with the same concentrations also in water.

The comparison between the curves in matrix and in water showed the absence of cortisol and its metabolites, and of matrix effect in urine. The matrix effect absence was also calculated comparing the peak areas of a standard solution at 100 ng mL^−1^ with the peak areas of the blank urine spiked after extraction with the same standard solution [38].

The limit of detection (LOD), defined as the lowest level at which a compound could be identified with a signal-to-noise (S/N) ratio greater than 3, and the limit of quantification (LOQ), defined as the lowest level at which a compound could be identified and quantified with a signal-to-noise ratio greater than 10, were calculated for each compound. For each corticosteroid, precision was determined by analyzing 3 QC samples at two different concentration levels (5 and 25 ng mL^−1^). Intra- and inter-assay precisions were expressed as CV%. Recovery (%) was calculated for all the compounds at two different urinary concentrations (5.0 and 25 ng mL^−1^). Robustness was observed in four different trials, fortifying blank urine samples at a concentration of 5.0 and 25 ng mL^−1^, changing slightly (± 10%) factors that may influence the outcome of the analysis. The factors were the volume of extraction solvent volume, centrifugation time, the volume of the mobile phase used for resuspension of dry extract and two different persons that performed analysis.

### Statistical analysis

Descriptive statistics, Kolmogorov Smirnov test for normality, and Mann Whitney test were performed using GraphPad InStat version 3.10 for Windows (GraphPad Software, San Diego, CA, USA).

## Results and discussion

### LC-MS^n^ analysis

In biological matrices, like human urine, LC–MS methods for corticosteroid imaging detection are usually performed in ESI ( +) using a triple quadrupole mass spectrometer operating in multiple reaction monitoring (MRM) acquisition mode [4, 39]. In the ESI positive ion mode, the precursor ions for fragmentation are either a pseudomolecular ion [M + H]^+^ or adducts with ammonium [M + NH_4_]^+^, methanol [M H + CH_3_OH]^+^, water [M + H − H_2_O]^+^, sodium [M + Na]^+^, etc. Generally, for structures with the carbonyl group on C3 position of steroid moiety the species [M + H]^+^ is used as precursor ion [15]).

In the ESI negative ion mode, we obtained a clear evidence that corticosteroids do not promptly generate the pseudomolecular ion[M − H]^−^ but a very stable adduct with formic acid. In the present work, 0.1% aqueous solution of formic acid was used as polar phase to form [M + HCOO]^−^, used as precursor ion for the MS^2^ fragmentation. The adduct fragmentation tends to generate a base peak as predominant while the abundance of the other products ions is less than 20% of the base peak. The most abundant product ion obtained in the MS^2^ analysis was used as precursor ion for MS^3^ analysis. For some molecules, it was also possible to perform MS^4^ experiments, as shown in Table 1. This possibility of performing consecutive MS^n^ acquisition steps presents a great advantage of the linear ion trap mass spectrometer with respect to common triple quadrupole mass spectrometer.

**Fig. 2:**
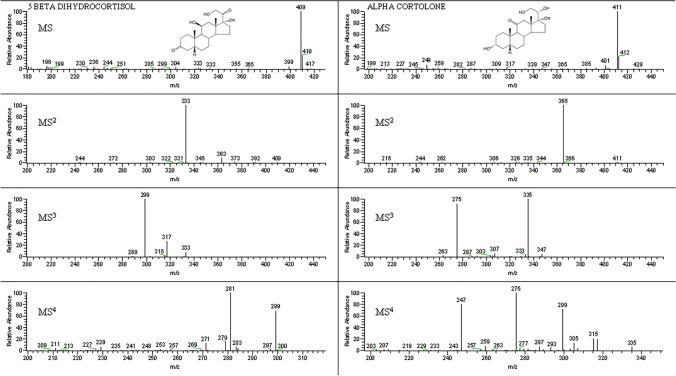
Full-scan spectra of the different MS^n^ steps for 5β-dihydrocortisol (represent C 20 keto compounds) and α-cortolone (to represent C 20 hydroxy compounds)

In Fig. 2, 5β-dihydrocortisol (to represent C 20 keto compounds) and α-cortolone (to represent C 20 hydroxy compounds) full-scan spectra of the different MS^n^ steps of analysis are reported as an example. In the MS^2^ full-scan spectra that comprise all parent ions, it is possible to observe two different types of fragmentations: all the compounds with a hydroxyl group in C20 (C20 hydroxy compounds), like α and β-cortolone, 20α and 20β-dihydrocortisol, and 20α and 20β-dihydrocortisone, give a very abundant and stable product ion corresponding to a loss of 46 Da (formic acid). All the compounds with a keto group on C20 (C20 keto series), such as 5α and 5β-dihydrocortisol, allotetrahydrocortisol and tetrahydrocortisol, allotetrahydrocortisone and tetrahydrocortisone, prednisolone, cortisol, and cortisone, give a very abundant and stable product ion corresponding to a loss of 76 Da (formic acid and formaldehyde CH_2_O). For this reason, also the internal standard methylprednisolone for the presence of the keto group on C20 (C20 keto compound), in the MS^2^ spectrum, gives a very abundant ion (m/z 343) corresponding to a loss of 76 Da from its adduct with formic acid in negative ion mode (m/z 419). For all the compounds, these abundant ions obtained after the first fragmentation (MS^2^) were used as precursor ions for MS^3^ analysis.

Due to the scarcity of product ions in MS^2^ spectra, MS^3^ analysis was chosen as the best compromise for specificity and sensitivity of the proposed method. Nevertheless, extreme specificity of MS^3^ with a very high *S*/*N* ratio allowed the achievement of an outstanding sensitivity. The differences in sensitivity operating in positive or negative mode were also checked working in MRM with a triple quadrupole, confirming the greater sensitivity of the negative mode for detection and quantification of glucocorticoids.

**Fig. 3:**
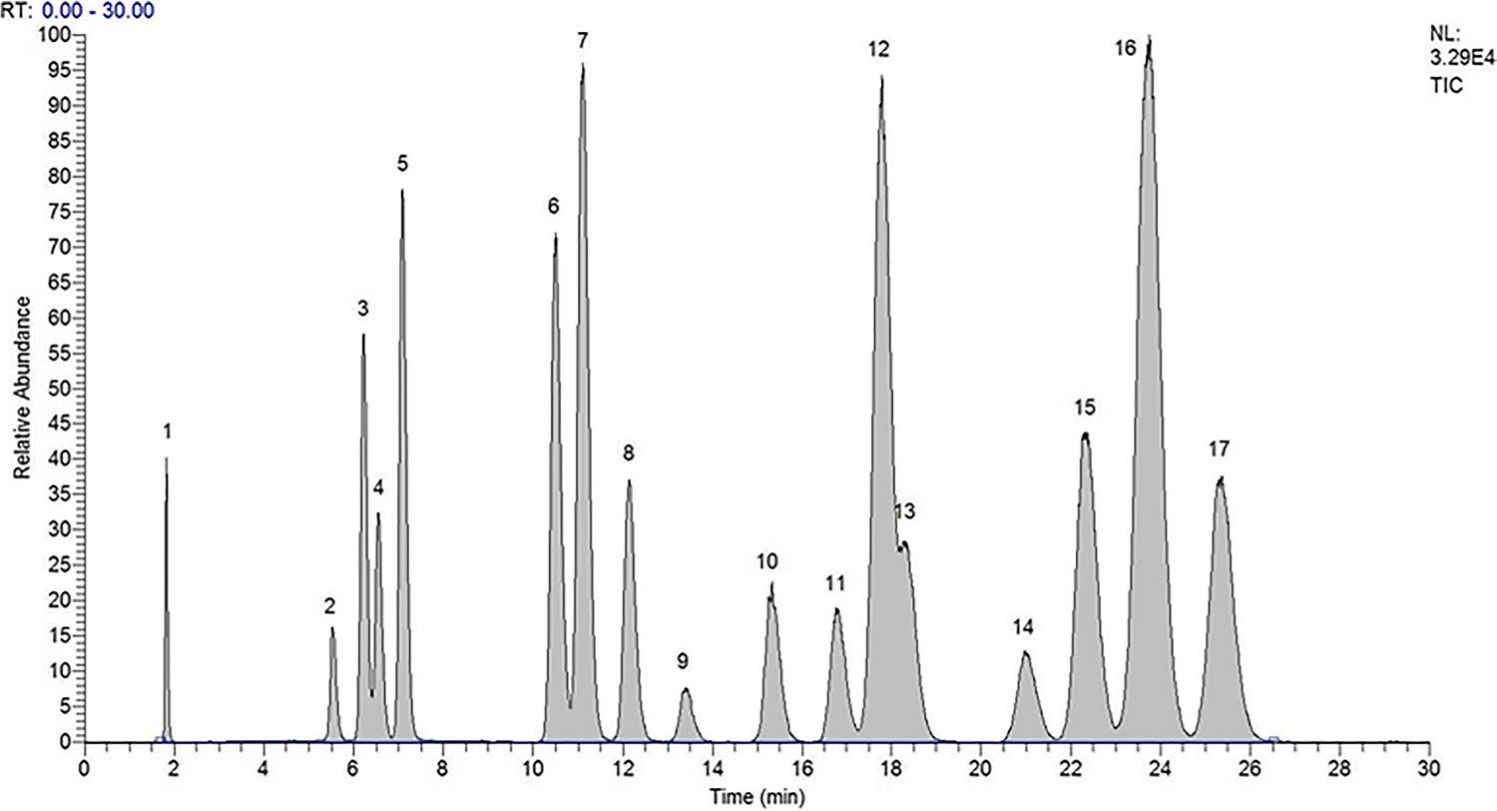
Full-scan LC-MS^3^ analysis of a blank human urine spiked at a concentration of 25 ng mL^−1^ with all the 16 compounds and IS. In order for retention time (Rt): 6β-hydroxycortisol (1), 20α-dihydrocortisol (2), 20α-dihydrocortisone (3), 20-β dihydrocortisol (4), 20β-dihydrocortisone (5), prednisolone (6), cortisol (7), cortisone (8), α-cortolone (9), β-cortolone (10), allotetrahydrocortisol (11), 5α-dihydrocortisol (12), tetrahydrocortisol (13), 6α-methylprednisolone as I.S. (14), allotetrahydrocortisone (15), 5β-dihydrocortisol (16), and tetrahydrocortisone (17)

Figure 3 shows an LC-MS^3^ analysis of a blank human urine spiked at a concentration of 25 ng mL^−^.^1^ with all the 16 compounds and IS. The chromatographic conditions were carefully optimized until they permitted the complete separation of the isomers [40]

### Method validation

Validation was performed according to WADA criteria [41] and is presented in Table 2. A correlation coefficient higher than 0.995 was obtained in both calibration curves (urine and water) for all the compounds except 6β-hydroxycortisol. This compound was discharged from validation because of its polarity that did not permit obtaining in liquid–liquid extraction a stable repartition between polar and organic phases. For each compound, the LOD was 0.01 ng mL^−1^ and the LOQ was 0.05 ng mL^−1^. Regarding cortisol these values are lower than those recently obtained with LC–MS/MS tandem mass spectrometry [12]. Intra- and inter-day precision (expressed as % CV) was in the range 1.4–9.2 and 3.6–10.4, respectively. Intra- and inter-day accuracies (expressed as % bias) ranged from 95 to 110 for all the analytes. Regarding the robustness of the method, none of the factors (volume of extraction solvent, centrifugation time, volume of resuspension solvent, persons that executed the sample preparation procedure) showed a significant variation in the concentration measurements.

**Table 2:** Analytical validation data for cortisol and its metabolites in human urine

Corticosteroid	LOD	LOQ	Conc	Recovery (bias)	Repeatability	
					Intra-day	Inter-day
	ng mL^−1^		ng mL^−1^	%	CV %	
20α-Dihydrocortisol	0.01	0.05	5.0	67	3.4	7.2
			25	73	3.5	9.2
20α-Dihydrocortisone	0.01	0.05	5.0	80	5.4	8.8
			25	81	4.3	7.5
20β-Dihydrocortisol	0.01	0.05	5.0	78	1.4	6.6
			25	82	9.2	7.0
20β-Dihydrocortisone	0.01	0.05	5.0	65	7.8	10.4
			25	75	7.4	10.2
Prednisolone	0.01	0.05	5.0	90	6.2	9.8
			25	95	3.4	8.9
Cortisol	0.01	0.05	5.0	89	1.8	4.5
			25	92	2.3	6.5
Cortisone	0.01	0.05	5.0	87	1.9	3.6
			25	91	3.5	3.6
α-Cortolone	0.01	0.05	5.0	88	4.2	3.3
			25	91	5.8	6.9
β-Cortolone	0.01	0.05	5.0	92	9.0	10.2
			25	95	8.2	8.1
Allo-tetrahydrocortisol	0.01	0.05	5.0	89	3.4	7.9
			25	92	5.5	10.2
5α-Dihydrocortisol	0.01	0.05	5.0	79	4.8	9.8
			25	88	4.9	9.3
Tetrahydrocortisol	0.01	0.05	5.0	90	1.9	8.7
			25	95	1.7	5.2
Allo-tetrahydrocortisone	0.01	0.05	5.0	88	8.2	9.0
			25	91	6.9	10.1
5β-Dihydrocortisol	0.01	0.05	5.0	88	7.2	10.2
				83	7.0	10.0
Tetrahydrocortisone	0.01	0.05	409	91	3.3	9.1
				95	4.9	5.7

Recoveries ranged from 65 to 95% at two different urinary concentrations (5.0 and 25 ng mL^−1^) for all corticosteroids were in line with data reported by others [39].The lowest recoveries were achieved for those compounds with the 20α or 20β-hydroxyl group (C 20 hydroxyl series). This polar chemical group could explain the lowest distribution in the organic phase during liquid–liquid extraction and consequently the lower recovery. Matrix effect was completely absent for all compounds working in ESI negative ion mode respect of the positive ion mode where the matrix effect influenced notably. Our validation data are comparable with those obtained by Wang et al. [42]. The authors reported that cortisol and metabolites were detected by means of high-resolution mass spectrometry with Orbitrap technology, which is recognized for its high specificity. Nevertheless, liner ion trap analysis preceded by almost complete chromatographical separation has exhibited a high specificity and sensitivity to candidate this methodology as a reliable alternative to high-resolution mass spectrometry (HRMS).

### Method application and results on healthy subjects

With the collection protocol adopted, all possible urine concentrations during the 24 h were obtained and the highest and the lowest levels for each molecule were determined. For all the free analytes in 50 healthy volunteers, the concentration range, expressed in nanograms per milliliter, is reported in Table 3. For the main compounds (cortisol and cortisone), the values found are in the line with recently reported concentration range [12, 15, 31]. The greatest concentration was detected for cortisone followed by cortisol. The 24-h urine from the two volunteers completely demonstrated the correlation of the circadian excretion profile of all the metabolites with the circadian rhythm of excretion of cortisol.

**Table 3:** Concentration range for all the free analytes in 50 healthy volunteers and concentrations for all the free analytes in Cushing patients, Addison patients, and doping cases

Corticosteroid	Fifty healthy people	Cushing patient 1	Cushing patient 2	Addison patient	Doping case 1	Doping case 2
	ng mL^−1^	ng mL^−1^	ng mL^−1^	ng mL^−1^	ng mL^−1^	ng mL^−1^
20α-Dihydrocortisol	5.00–50.0	939	115	0.30	0.50	1.00
20α-Dihydrocortisone	2.50–37.0	89.0	60.0	2.00	1.00	1.00
20β-Dihydrocortisol	3.00–45.0	142	58.0	0.50	1.00	1.00
20β-Dihydrocortisone	1.50–21.0	10.0	15.0	0.10	nd	1.00
Prednisolone	0.05–1.00	9.0	3.00	nd	nd	0.05
Cortisol	4.00–71.0	364	138	0.50	1.00	4.00
Cortisone	5.00–76.0	112	80.0	3.50	3.00	5.00
α-Cortolone	1.00–24.0	140	40.0	2.50	1.50	1.00
β-Cortolone	1.00–9.00	38.0	19.0	5.00	1.00	1.00
Allo-tetrahydrocortisol	0.05–3.00	7.0	9.00	0.20	nd	nd
5α-Dihydrocortisol	0.05–5.00	0.1	0.30	nd	nd	nd
Tetrahydrocortisol	3.00–46.0	459	111	10.0	2.00	3.00
Allo-tetrahydrocortisone	0.05–2.00	1.0	4.5	nd	nd	nd
5β-Dihydrocortisol	0.05–0.20	2.0	0.5	nd	nd	nd
Tetrahydrocortisone	2.00–37.0	135	79.0	12.0	5.00	2.00

*nd* not detected

**Fig. 4:**
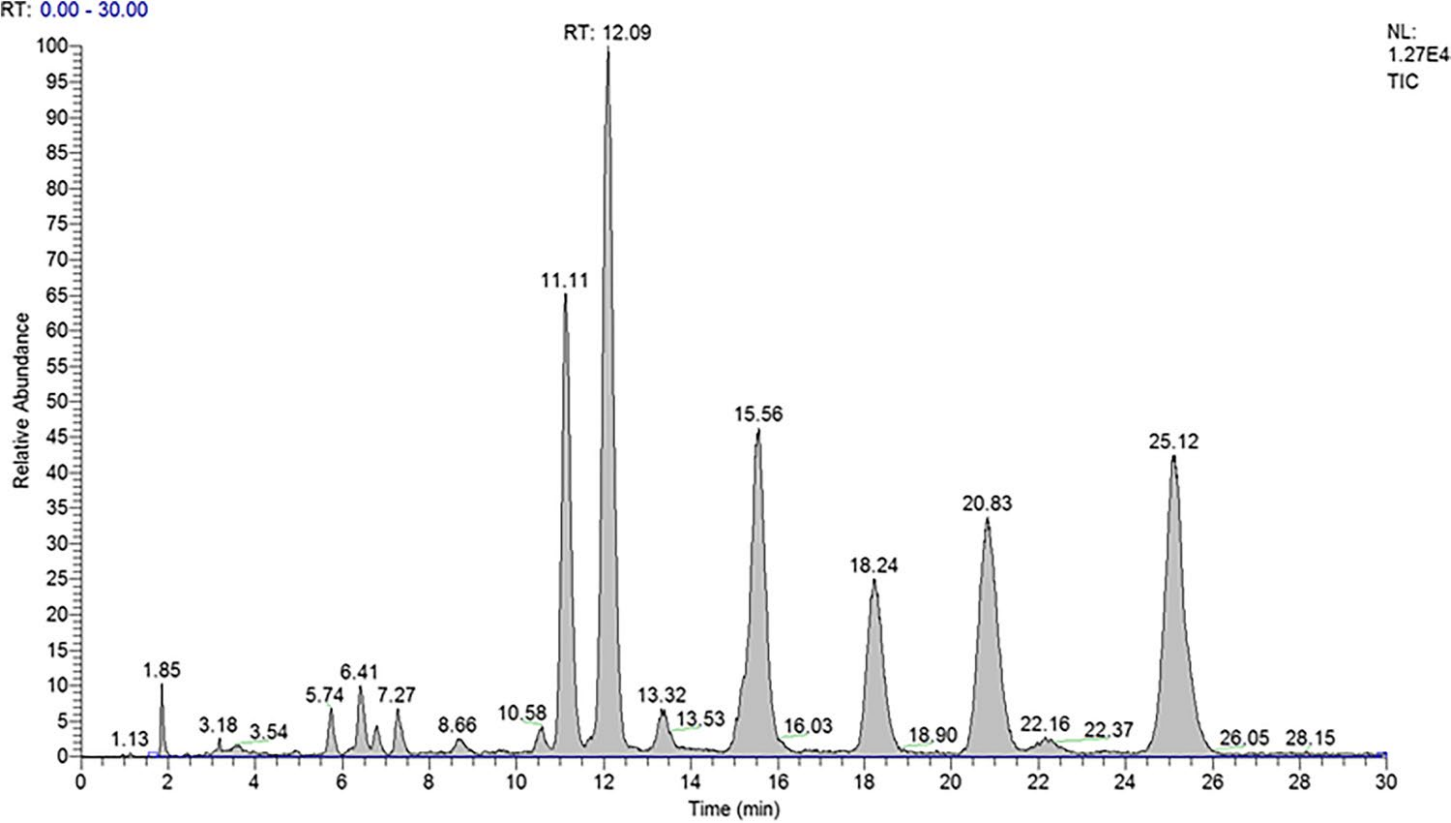
Full-scan LC-MS^3^ analysis of a human urine after liquid–liquid extraction

The normal distribution for steroid concentration in male and female urine was checked through the Kolmogorov Smirnov test, and, due to the lack of it in most of the groups, the Mann–Whitney test for unpaired samples was performed to check for differences between male and female urine. No difference (*P* > 0.05) was observed in urinary concentrations for all the steroids, between male and female. The total ion current (TIC) of an LC-MS^3^ analysis of a human urine after liquid–liquid extraction is shown in Fig. 4.

The percentage estimation of the conjugated form with glucuronic acid of all the compounds was performed on 20 urine samples (10 males, 10 females).

**Fig. 5:**
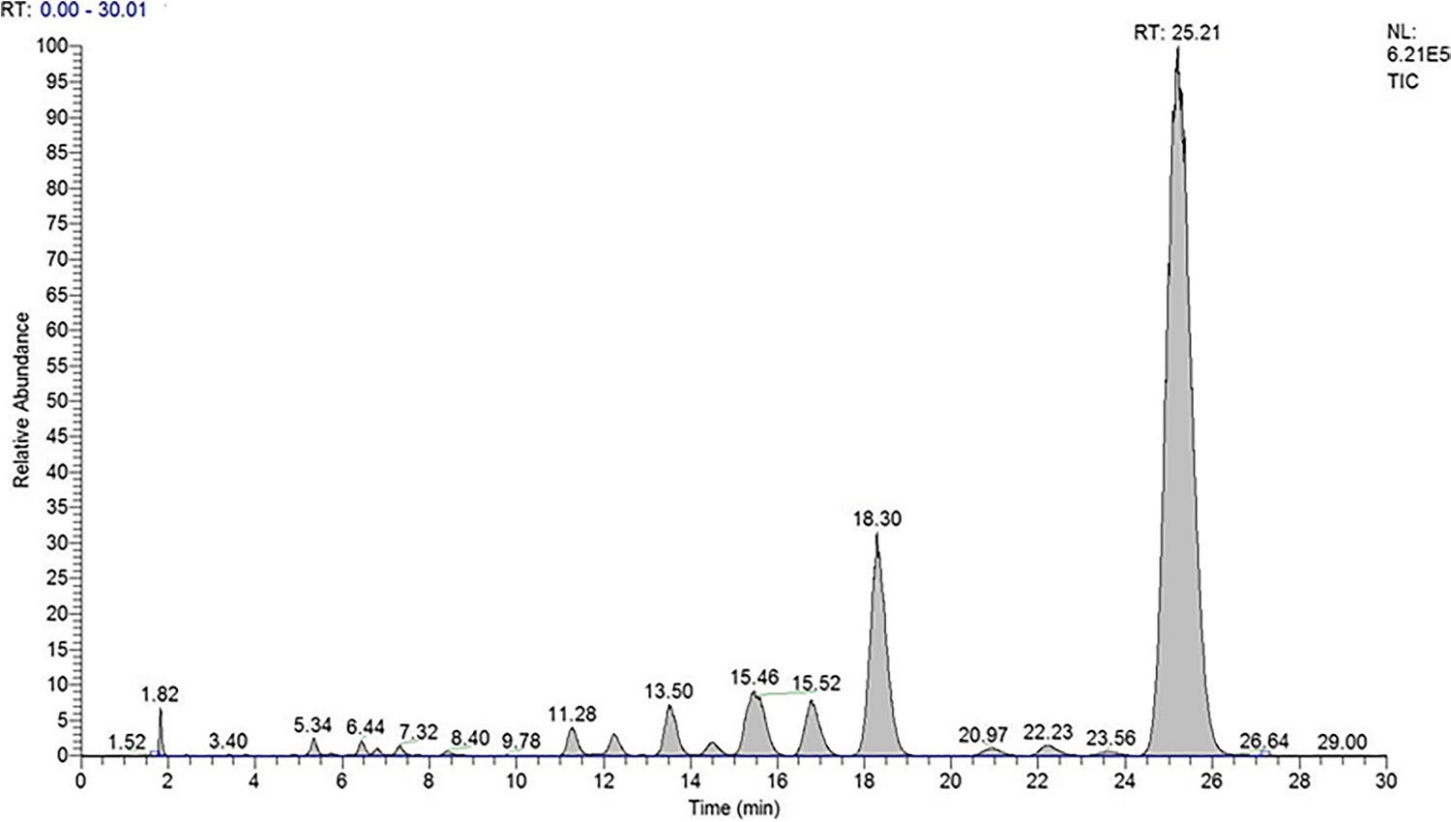
Full-scan LC-MS^3^ analysis of a human urine after hydrolysis step and liquid–liquid extraction

The concentrations of some conjugated metabolites, such as tetrahydrocortisol and tetrahydrocortisone, α-cortolone, and allotetrahydrocortisol, were higher than the highest calibration curve level (500 ng mL^−1^) used to calculate the free form. Therefore, a semiquantitative approach was applied, because the study aimed at the quantitation of the free forms, just to bypass the hydrolysis step, in a future routine procedure. After the hydrolysis step, tetrahydrocortisone and tetrahydrocortisol showed the highest urinary concentrations. The total ion current (TIC) of an LC-MS^3^ analysis of a human urine after the hydrolysis step is shown in Fig. 5.

About conjugation percentage, we experimentally observed 30–70% range of the glucuronated forms of cortisol, cortisone, 20α-dihydrocortisol, 20-β dihydrocortisol, 20α-dihydrocortisone, and 20β-dihydrocortisone. These molecules, on the A ring of the cyclopentanoperhydrophenanthrene nucleus, are characterized by a C4–C5 double bond and by the presence of a C3-keto group. On the other hand, a higher glucuronidation percentage, near to 100%, was calculated for allotetrahydrocortisol, tetrahydrocortisol, allotethrahydrocortisone, tetrahydrocortisone, α-cortolone, and β-cortolone. In these molecules, a α-hydroxyl group is bound to C3 of the A ring. This group chemically is favored for glucuronic acid conjugation by UDP glucuronyl transferase enzyme.

It is important to note that, considering a urine average volume of 1.5 L produced in 24 h, our computed concentrations agreed with the values expressed in micrograms per day reported by other authors [17].

#### Clinical application

To evaluate the possible application of the method in clinical diagnosis, two urine samples from Cushing patients and one sample from an Addison patient were analyzed.

Cushing’s syndrome is characterized by chronic glucocorticoid excess. It can be adrenocorticotropic hormone (ACTH) dependent (Cushing’s disease or ectopic ACTH production) or ACTH independent (adrenal adenoma or carcinoma). In patients with this syndrome, cortisol concentration is higher than in healthy people [7]. Consequently, also, the cortisol metabolite concentration is usually expected to be high [10]. Without any kind of clinical treatment, Cushing effect syndrome may include osteoporosis, hypertension, type 2 diabetes, frequent or unusual infections, and muscle mass loss and strength [6]. The diagnosis is really complex, and a lot of techniques are used. The recommended screening test is an overnight dexamethasone suppression test or a 24-h urinary free cortisol collection quantification [43].

Addison’s disease, also known as primary adrenal insufficiency and hypocortisolism, is a long-term disease characterized by reduced production of steroid hormones. Symptoms may include abdominal pain, weakness, and weight loss. Under certain circumstances, symptoms such as low blood pressure, vomiting, lower back pain, and loss of consciousness occur. An adrenal crisis can be triggered by stress, such as from an injury, surgery, or infection. Death may occur without treatment. The diagnosis made by biochemical laboratory tests in which blood and/or urine cortisol levels are measured before and after a synthetic form of ACTH is given by injection [44]. In urine the cortisol concentration is expected to be extremely low and consequently also all its metabolite concentration.

The urinary corticosteroid concentrations of tree patients (two with Cushing’s syndrome and one with Addison’s disease) are presented in the Table 3. The revealed concentrations clearly showed the macroscopic differences between healthy and pathological conditions. In the two Cushing urine samples, it was undoubtedly evident the overproduction of cortisol and of the metabolites with respect the healthy subjects. In the urine from the Addison patient, we obtained the opposite situation with an evident suppression of cortisol and metabolites with respect to the concentrations calculated in healthy people.

It is clearly evident that it is possible to select other potential biomarkers, in addition to cortisol, effective in recognizing a non-physiological situation. As defined by Kushnir et al. [16]: “biochemicals markers are endogenous compounds that are either not present in a normal physiological state or present within certain range of concentrations (e.g. intermediate and product of metabolic pathways).” Among these, “prednisolone” was also included. It was demonstrated that this molecule, very commonly used in therapy as a synthetic corticosteroid, has furthermore an endogenous production related to cortisol metabolism. In a normal subject, in urine, its concentration is usually lower than 0.5 ng mL^−1^. It was moreover possible to observe that, in Cushing patients, the calculated values were higher than the highest value (1 ng mL^−1^) calculated on 50 healthy subjects’ urine. This evidence can be considered as an additional confirmation of our study [34], about its endogenous origin and correlation with cortisol metabolism. Its high level in a pathological condition makes also prednisolone a candidate biomarker.

#### Antidoping application

In the World Anti-Doping Agency (WADA’s) list, glucocorticoids (e.g., prednisolone, dexamethasone, etc.) are considered doping substances and they are prohibited for the in-competition sport activities. Nevertheless, they are frequently abused due to their common utilization as anti-inflammatory drugs. The consumption of these drugs prompts a negative feedback inhibition of hypothalamus-hypophysis-adrenal axis, causing a lower production of cortisol and consequently of all its metabolites. When used in doping purposes, usually, they are given in small doses over a relatively long time period [45]. Because of their low concentrations and the fact that there is only limited understanding of their metabolism, the determination of exogenously administered corticosteroids remains difficult and rather problematic. The question that arises is whether the absence of these substances actually means that the subject had not abused them? An answer to this question cannot be given with certainty; studies on the impact of synthetic analogs on the metabolism of endogenous glucocorticosteroids would perhaps provide some explanations. Therefore, the accurate and precise measurement of endogenous corticosteroids can be considered a powerful tool in the identification of illegal glucocorticoid treatments. For this reason, the here-described technique for screening of cortisol and its metabolites can be used in antidoping analysis [42].

To test the feasibility of our method, two different human urine samples aimed for the antidoping control were analyzed. Those two samples were chosen appropriately, as the presence of betamethasone and dexamethasone was confirmed previously. In Table 3, the concentrations of each compound calculated in those two urine samples are reported. Doping case 1 shows a clear suppression of all steroids while in case 2, 20α-dihydrocortisol, 20α-dihydrocortisone, 20β-dihydrocortisol, and 20β-dihydrocortisone underwent a suppression, with other steroids in the low range value. It is therefore evident that biomarkers, different from cortisol and revealed by our method, could be useful to identify a pharmacologic treatment.

## Conclusion

In this study, an LC-MS^n^ method for simultaneous quantification of urinary cortisol and its 15 metabolites in human urine was developed and validated. The excellent sensitivity, accompanied by extreme specificity due to complete chromatographical separation of all species with subsequent detection in ESI negative ion mode with a linear ion trap like analyzer, undoubtedly showed the suitability of this method for different purposes. The method was used to calculate the concentration range of all free compounds in human urine of 50 healthy subjects and found its application in the identification of pathological conditions in which the corticosteroid metabolism is involved (ex. Cushing and Addison disease), as well as to identify urine of a sportsmen doped with corticosteroids.

The method evidently shows a high potential for diagnostics as only 2 mL urine from a single sampling was needed instead of the 24-h collection, commonly used for the assay of free cortisol in urine. Therefore, especially for its potential clinical application, it could be considered as a rapid screening tool, followed in a second time by further medical diagnostic investigations.

Moreover, the possibility of using cortisol metabolites as specific biomarkers of cortisol metabolism disbalance allows solving and bypassing some analytical problems that can be related to qualitative and quantitative analyses of cortisol alone [46, 47]. It remains to perform the studies on a huge number of human urines deriving from subjects with various pathophysiological conditions caused by disturbances and lack of regulation in cortisol metabolism.

## Declarations

Human urine was collected anonymously for analytical method validation from informed volunteers. The urine sample collection was approved by the medical director of Unirelab srl on October 2019.
